# Supplementary figures and images for: Interplay between the cell envelope and mobile genetic elements shapes gene flow in populations of the nosocomial pathogen Klebsiella pneumoniae
Source: PLoS Biol. 2021 Jul 6;19(7):e3001276. doi: 10.1371/journal.pbio.3001276 (PMC8259999; doi:10.1371/journal.pbio.3001276)

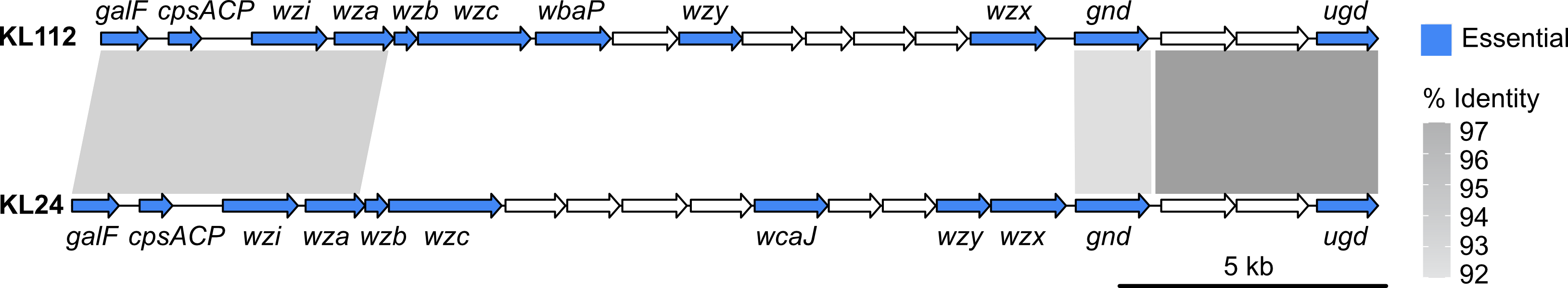

Supplement: S1 Fig — Two CLTs (KL112 and KL24) involved in CLT swap, with the essential genes for capsule expression colored in blue. Gray tracks correspond to the sequence identity (computed using blastn) above 90% (see scale) to indicate highly similar homologs (liable to recombine). CLT, capsular locus type. (TIFF) [file pbio.3001276.s001.tiff]

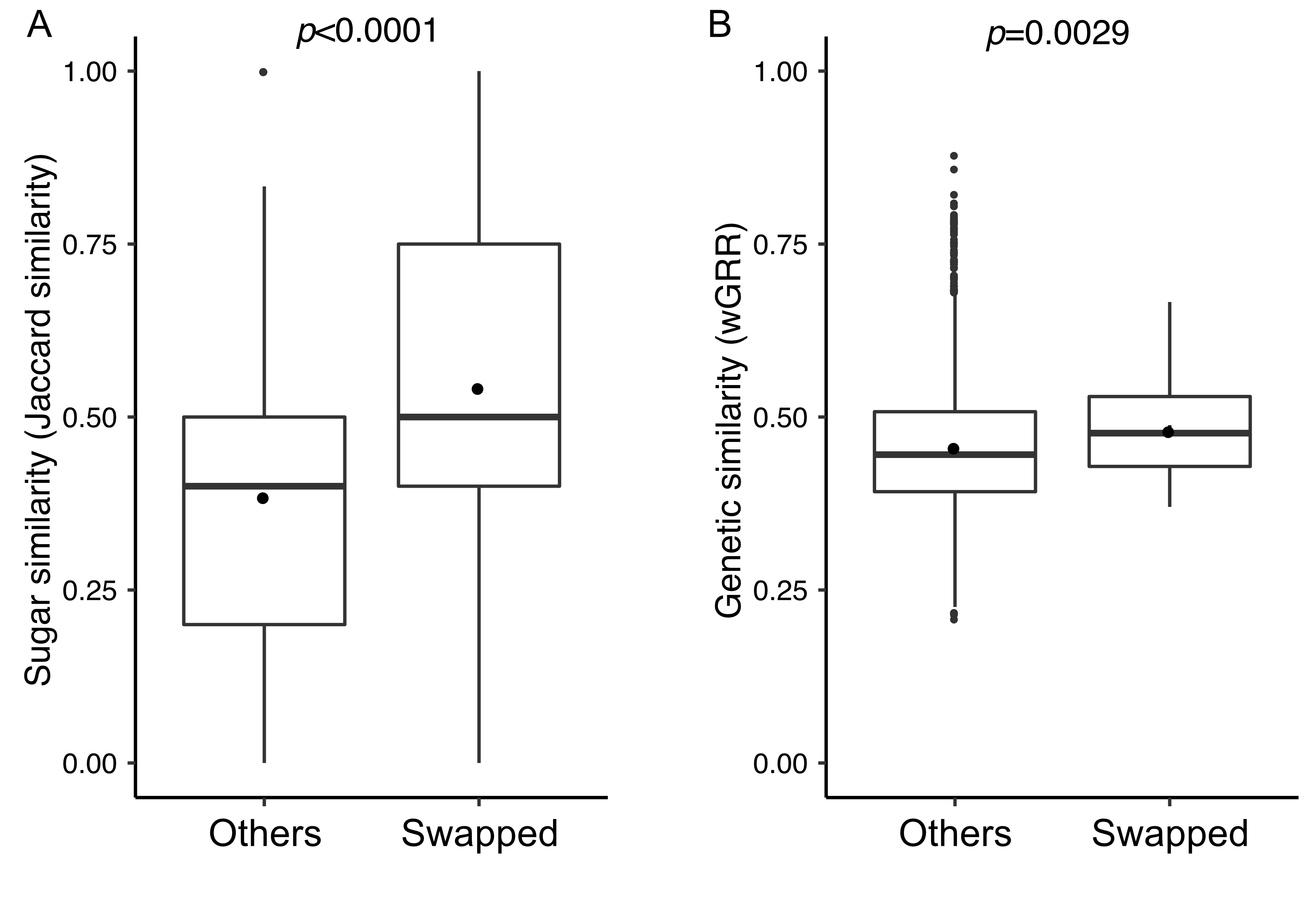

Supplement: S2 Fig — (A) Comparison of sugar composition similarity (Jaccard similarity) between swapped vs. others CLTs. (B) Comparison of genetic similarity (wGRR) between swapped vs. others CLTs. The p-value displayed is for the 2-sample Wilcoxon test (https://doi.org/10.6084/m9.figshare.14673180). CLT, capsular locus type; wGRR, gene repertoire relatedness weighted by sequence identity. (TIFF) [file pbio.3001276.s002.tiff]

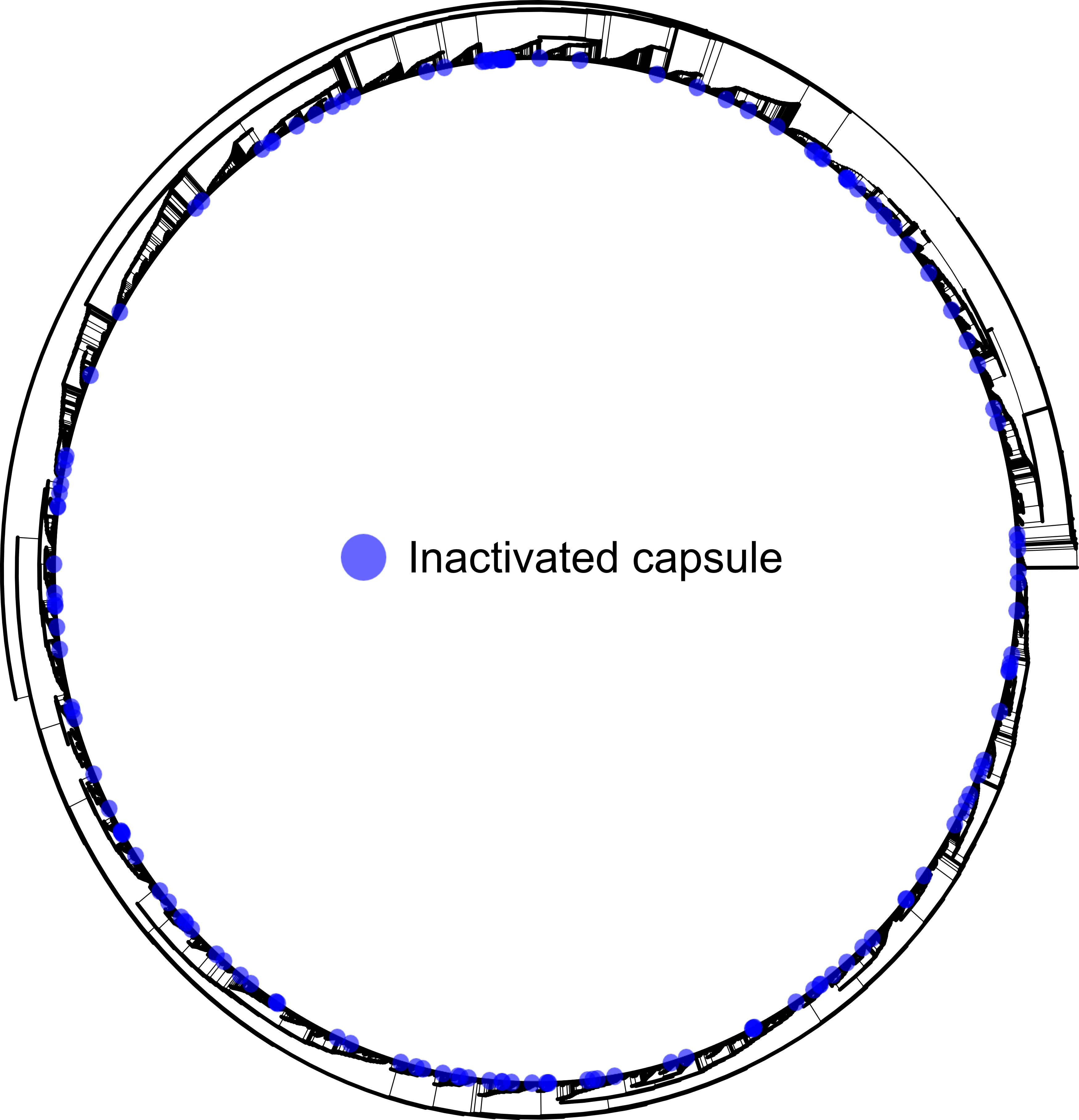

Supplement: S3 Fig — The blue dots represent the putative inactivated capsules, which have at least 1 essential gene for capsule production pseudogenized or deleted (https://doi.org/10.6084/m9.figshare.14673156). (TIFF) [file pbio.3001276.s003.tiff]

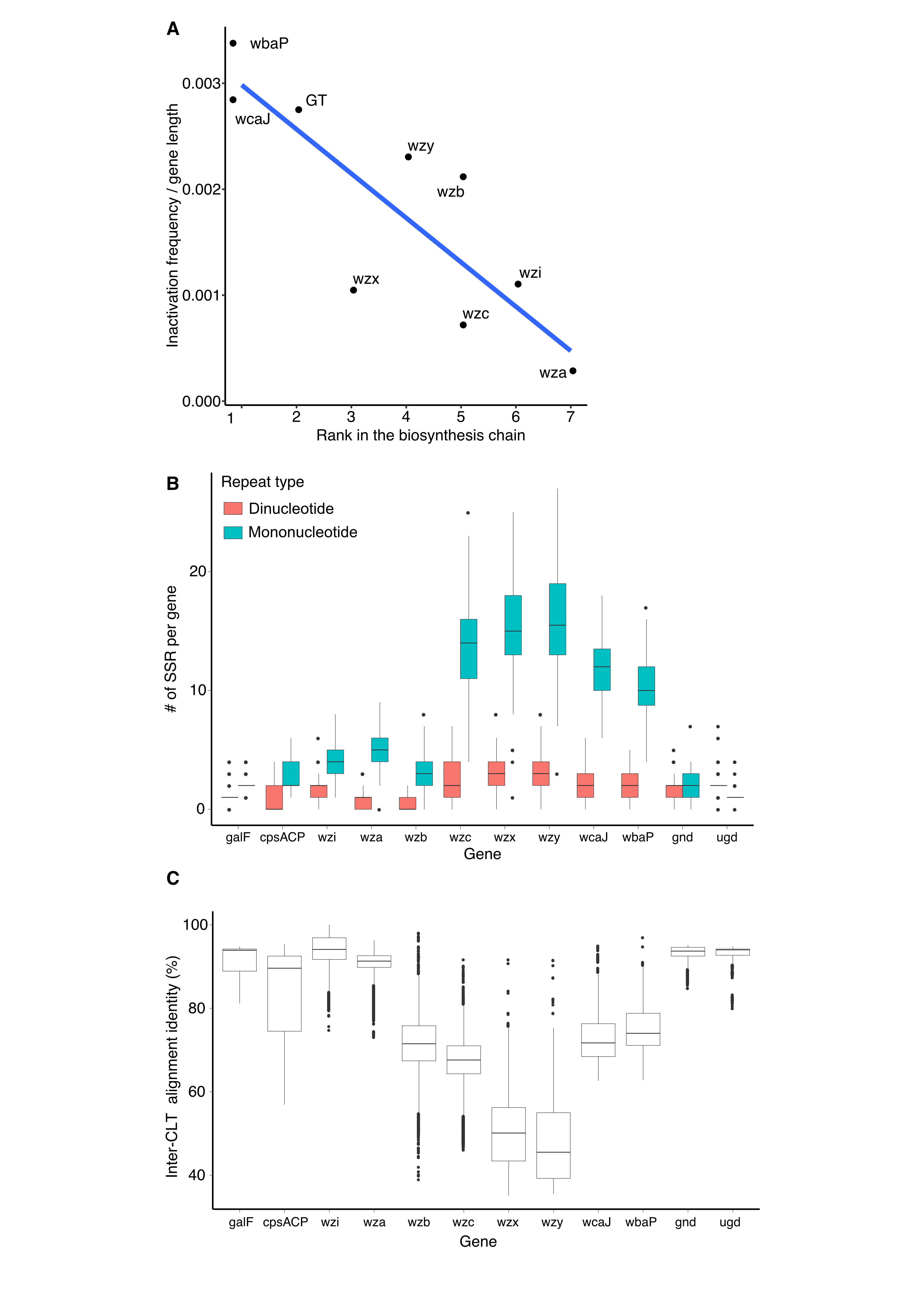

Supplement: S4 Fig — (A) Linear regression between the inactivation frequency normalized by average gene length and the rank of each gene in the biosynthesis pathway (p = 0.005, R2 = 0.7) (https://doi.org/10.6084/m9.figshare.14673183). (B) Number of SSR in the core capsule genes in the Kaptive reference database (https://doi.org/10.6084/m9.figshare.14673174). (C) Genetic diversity of core capsule genes within the Kaptive reference database, represented by the percent of identity of all pairwise alignments of the proteins from different reference capsule loci (https://doi.org/10.6084/m9.figshare.14673192). SSR, simple sequence repeats. (TIFF) [file pbio.3001276.s004.tiff]

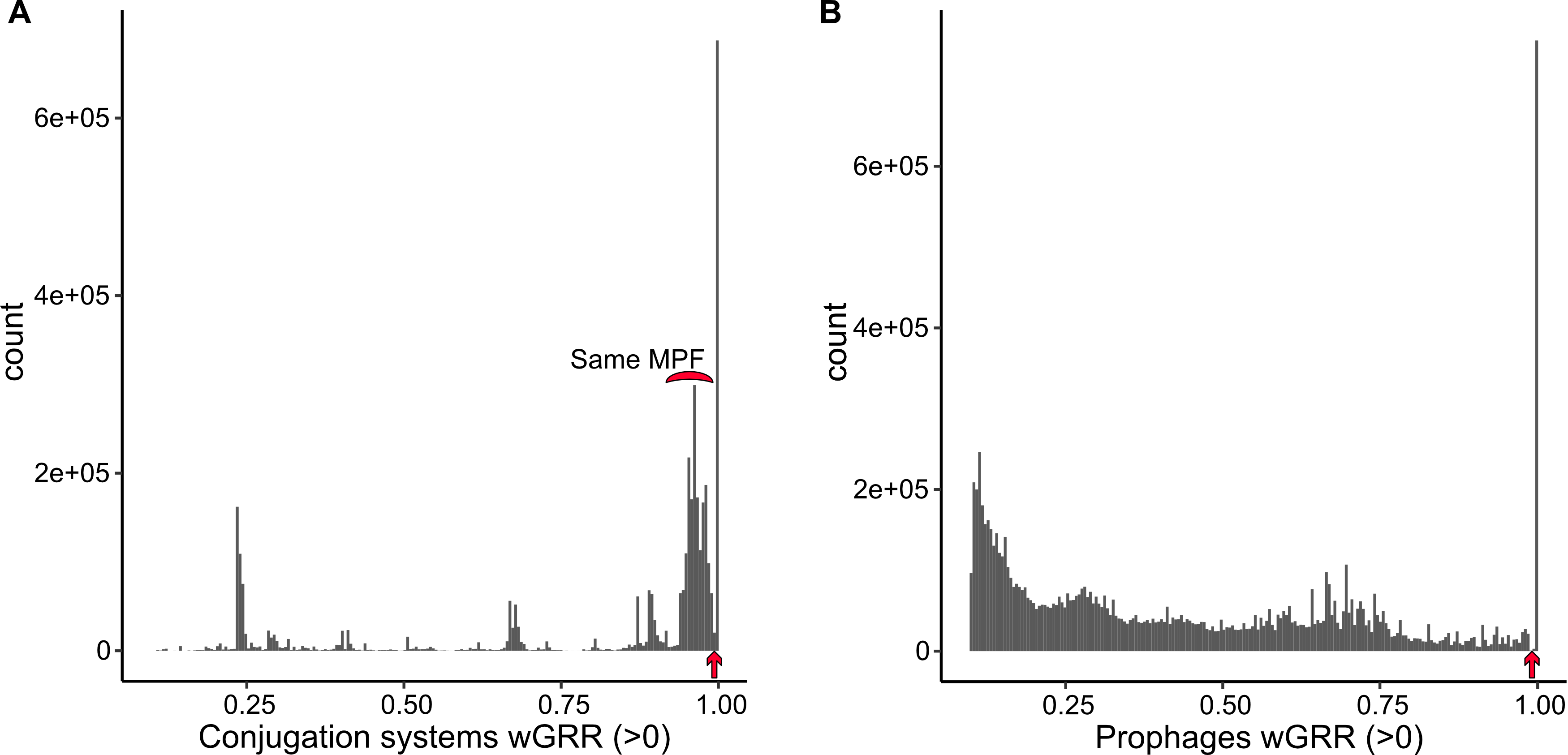

Supplement: S5 Fig — Distribution of the similarity measured by wGRR between pairs of conjugative systems (A) and between pairs of prophages (B) for wGRR >0. The arrows represent the threshold (wGRR >0.99) set for clustering into families of highly similar elements. Since we performed transitive clustering to build the families, some elements belonging to the same families have wGRR <0.99. We annotated the distribution of conjugation systems belonging to the same MPF type, which shows that systems of the same MPF are very similar but are below the selected threshold for clustering (https://doi.org/10.6084/m9.figshare.14673144 and https://doi.org/10.6084/m9.figshare.14673186). MPF, mating pair formation; wGRR, gene repertoire relatedness weighted by sequence identity. (TIFF) [file pbio.3001276.s005.tiff]

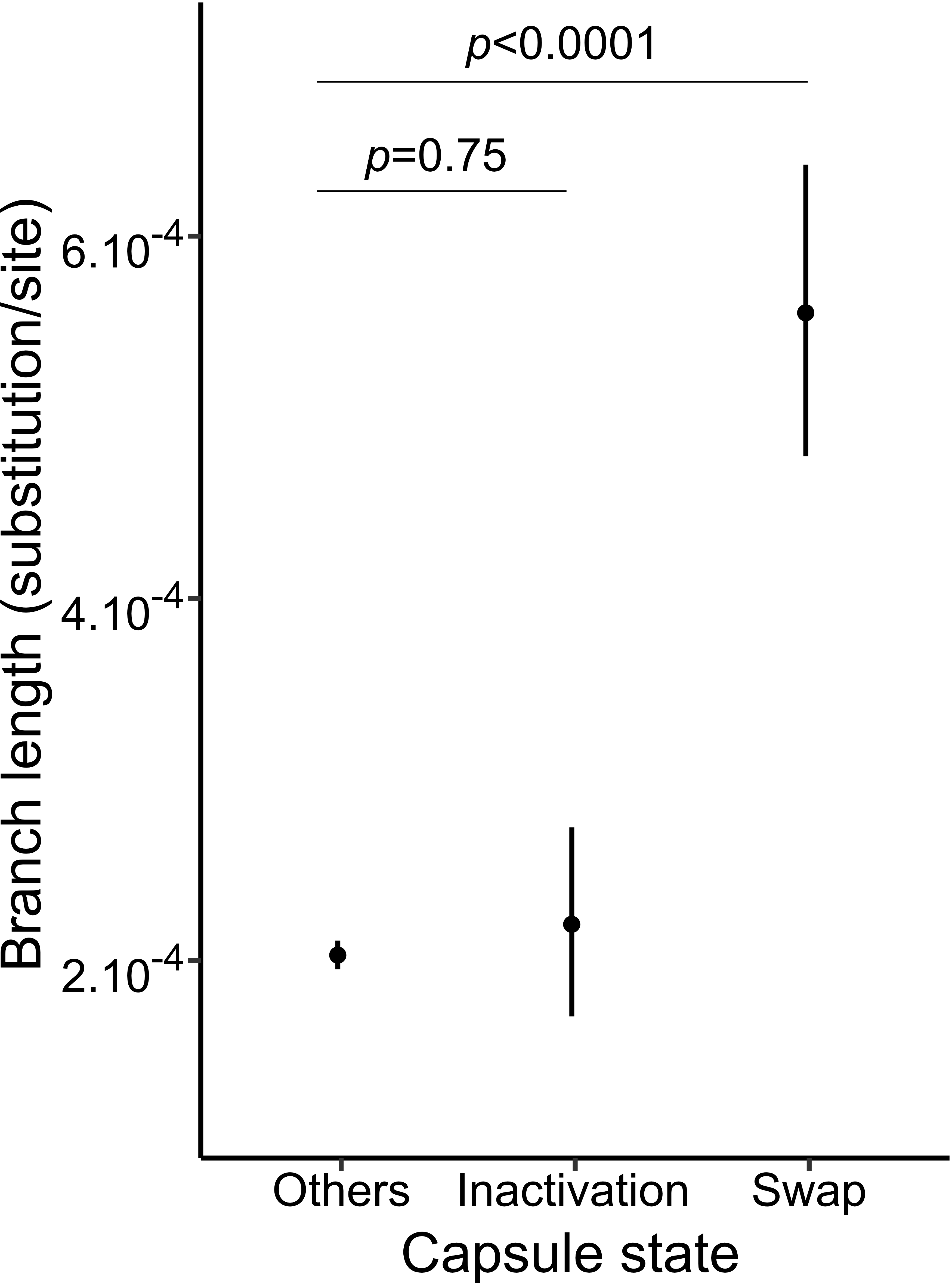

Supplement: S6 Fig — The capsule state changes among branches of the species tree are represented on the x axis, and the branch length is represented on the y axis in substitution per site. Individual points represent the mean for each group, and the bars represent the standard error. The p-values for the t test are represented on top of each comparisons (https://doi.org/10.6084/m9.figshare.14673159). We also performed a 2-sample Wilcoxon test to compare the medians (“Others” vs. “inactivation”: p < 0.0001; “Others” vs. “Swap”: p < 0.0001). (TIFF) [file pbio.3001276.s006.tiff]

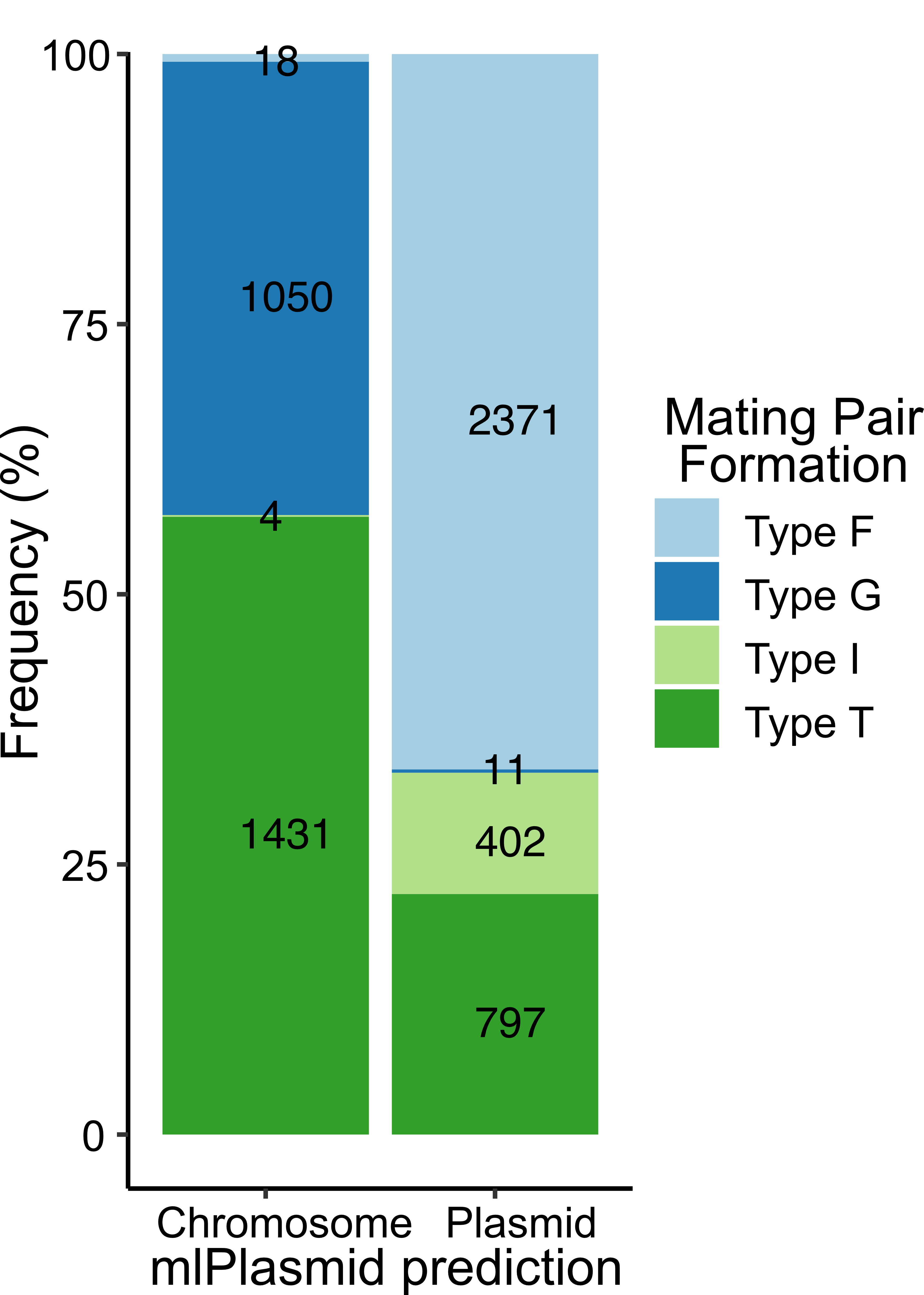

Supplement: S7 Fig — Conjugation systems are classified in 2 categories according to their genomic location, which was predicted with the mlplasmids classifier. The MPF was predicted with the CONJscan module of MacSyfinder. Absolute number of systems are displayed for each category (https://doi.org/10.6084/m9.figshare.14673189). MPF, mating pair formation. (TIFF) [file pbio.3001276.s007.tiff]

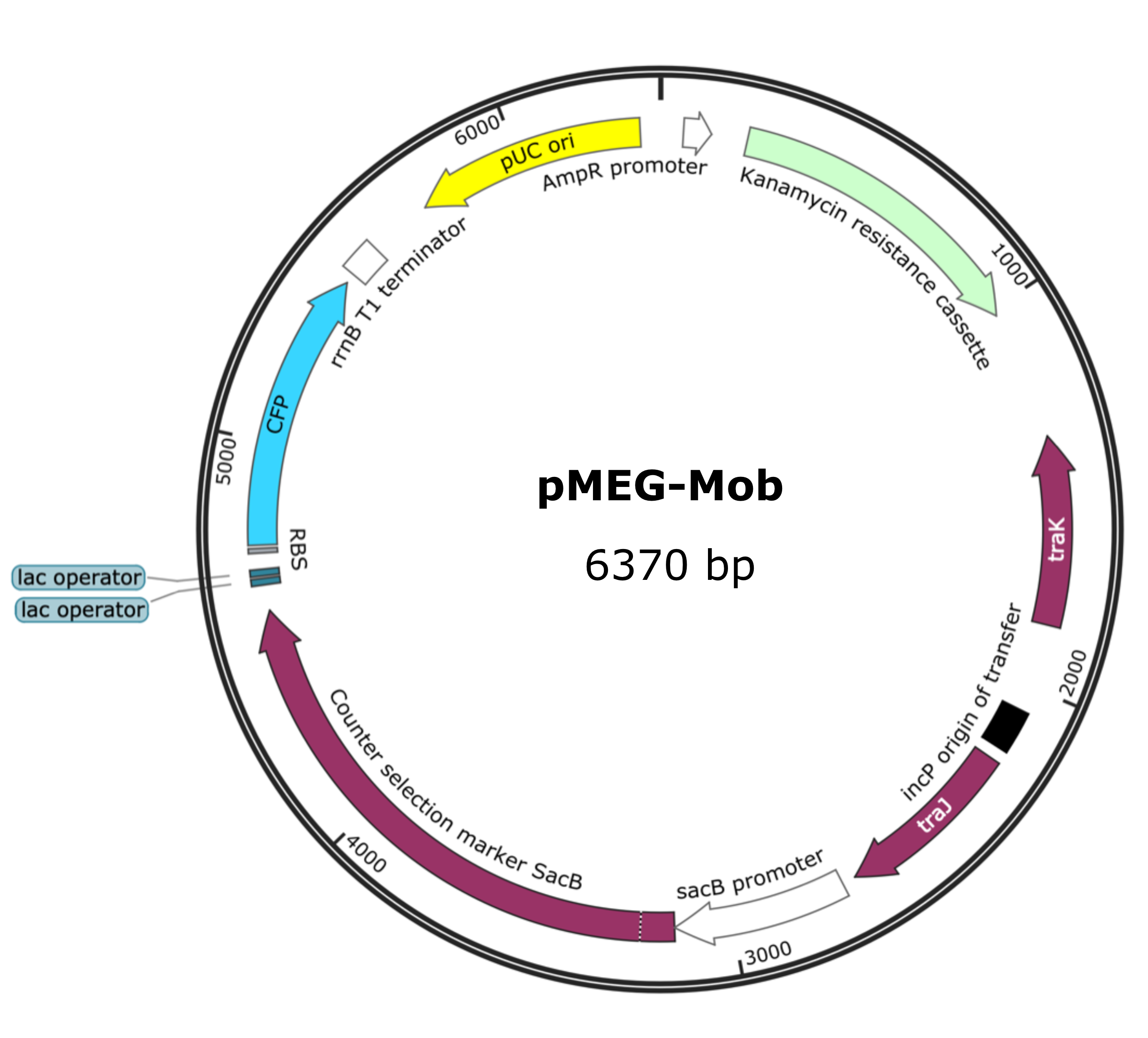

Supplement: S8 Fig — pMEG-Mob was constructed by Gibson assembly from plasmids pKNG101 and pZE12. It encodes a colE1/pUC origin of replication (high copy number), a selectable marker (Kanamycin resistance cassette, green), the mobilizable region of pKNG101 which is composed of the origin of transfer of RK2 and 2 genes involved in conjugation (traJ and traK), a counter selectable marker (sacB), and an inducible CFP gene (IPTG induction). pMEG-Mob can only be mobilized in trans and thus can only be transferred from a strain expressing the RK2 conjugative machinery, which is absent from the panel of strains we used as recipients. (TIFF) [file pbio.3001276.s008.tiff]
