# Supplementary material for: Interplay between the cell envelope and mobile genetic elements shapes gene flow in populations of the nosocomial pathogen Klebsiella pneumoniae
Source: PLoS Biol. 2021 Jul 6;19(7):e3001276. doi: 10.1371/journal.pbio.3001276 (PMC8259999; doi:10.1371/journal.pbio.3001276)
Supplement: S1 Text — Supporting information containing detailed list of essential capsule genes, strains, plasmids, and primers used in this study. (PDF) [file pbio.3001276.s009.pdf]

S1 Text

Table A. Essential genes for capsule production. The order corresponds to the order in the biosynthesis chain. The references correspond to published studies providing experimental evidence that these genes are essential for capsule production. ND stands for undetermined.

**Gene name**	**Order**	**Function**	**Reference**
galF	ND	UTP-glucose-1-phosphate uridylyl transferase	[1–3]
cpsACP	ND	Acid phosphatase homolog	[2,4]
wza	7	Outer membrane export protein	[2,4–6]
wzb	5	Protein-tyrosine phosphatase	[2,4,6]
wzc	5	Protein-tyrosine kinase	[2,4,6]
wzy	4	Capsule repeat-unit polymerase	[2,4,6]
wzx	3	Flippase	[2,4,6,7]
wzi	6	Outer membrane protein, surface assembly of capsule	[2,4,6]
wcaJ	1	Undecaprenyl-phosphate glucose phosphotransferase, initiating glycosyltransferase	[2,4–6,8]
wbaP	1	Undecaprenyl-phosphate galactose phosphotransferase, initiating glycosyltransferase	[2,4–6]
gnd	ND	6-phosphogluconate dehydrogenase	[1,2]

Table B. Strains used in this study

Strain number	Strain name	Species	ST	Capsule locus type	Country	Isolation	Accession
24	342	*K. variicola*	ST146	KL30	USA	Corn	GCF_001913175.1
26	BJ1	*K. pneumoniae*	ST380	KL2	France	Liver abscess	GCF_900978065.1
56	NTUH K2044	*K. pneumoniae*	ST23	KL1	Taiwan	Liver abscess	GCF_000009885.1
58	SB4454 – CG43	*K. pneumoniae*	ST86	KL2	Taiwan	Liver abscess	NC_022566, NC_005249, SAMEA2633716
63	NJST258-1	*K. pneumoniae*	ST258	KL107	USA	Urinary tract	GCF_000598005.1
208	SB32	*K. pneumoniae*	ST20	KL111	Germany	Blood	SAMEA8547061
210	CIP 52.229 - SB3260	*K. pneumoniae*	ST59	KL24	NA	NA	SAMEA8547063
212	SB5199	*K. pneumoniae*	ST2435	KL30	France	Poultry (carriage)	SAMEA8547062
213	SB5701	*K. pneumoniae*	ST16	KL107	Cambodia		SAMEA5753389
100	*E. coli S17* MFD λpir	*E. coli*				Laboratory strain	[9]
287	*E. coli* DH5α λpir	*E. coli*				Laboratory strain	

Table C. Plasmids used in this study

Plasmid name	Resistance	Reference
pKNG101	Tet	[10]
pZE12-CFP	Km	[11]
pMEG-Mob	Km	This study

Table D. Primers used for pMEG-Mob construction

Primer name	Direction	Sequence
pKNG_pCONJ-R	Reverse	GACGAAAGGGCCTCGTGATAGAGGCCGGGTTAAGAGTT
pZE12_pCONJ-R	Reverse	ACCCAAACAGTAGAATTCCCTCCCTTAACGTGAGTTTTCGTTC
pKNG_pCONJ-F	Forward	CGAAAACTCACGTTAAGGGAGGGAATTCTACTGTTTGGGTGT
pZE12_pCONJ-F	Forward	CCAACTCTTAACCCGGCCTCTATCACGAGGCCCTTTCGTC
pCONJ_verif-F	Forward	GATGGCTACCAAGGCGAAGAA
pCONJ_verif-R	Reverse	CTCGCCGCAGCCGAACGCCTAG

References for S1 Text

1. Rafał G. Heterogeneity of galF and gnd of the cps region for capsule synthesis in clinical isolates of Klebsiella pneumoniae. Pol J Microbiol. 2007;56: 83–88.

2. Dorman MJ, Feltwell T, Goulding DA, Parkhill J, Short FL. The Capsule Regulatory Network of Klebsiella pneumoniae Defined by density-TraDISort. mBio. 2018;9: e01863-18. doi:10.1128/mBio.01863-18

3. Peng D, Li X, Liu P, Zhou X, Luo M, Su K, et al. Transcriptional regulation of galF by RcsAB affects capsular polysaccharide formation in Klebsiella pneumoniae NTUH-K2044. Microbiol Res. 2018;216: 70–78. doi:10.1016/j.micres.2018.08.010

4. Lin C-L, Chen F-H, Huang L-Y, Chang J-C, Chen J-H, Tsai Y-K, et al. Effect in virulence of switching conserved homologous capsular polysaccharide genes from Klebsiella pneumoniae serotype K1 into K20. Virulence. 2017;8: 487–493. doi:10.1080/21505594.2016.1228508

5. Buffet A, Rocha EPC, Rendueles O. Selection for the bacterial capsule in the absence of biotic and abiotic aggressions depends on growth conditions. bioRxiv. 2020 [cited 21 Aug 2020]. doi:10.1101/2020.04.27.059774

6. Tan YH, Chen Y, Chu WHW, Sham L-T, Gan Y-H. Cell envelope defects of different capsule-null mutants in K1 hypervirulent Klebsiella pneumoniae can affect bacterial pathogenesis. Mol Microbiol. 2020;113: 889–905. doi:10.1111/mmi.14447

7. Anderson MT, Mitchell LA, Zhao L, Mobley HLT. Capsule Production and Glucose Metabolism Dictate Fitness during Serratia marcescens Bacteremia. mBio. 2017;8: e00740-17. doi:10.1128/mBio.00740-17

8. Tan D, Zhang Y, Qin J, Le S, Gu J, Chen L, et al. A Frameshift Mutation in wcaJ Associated with Phage Resistance in Klebsiella pneumoniae. Microorganisms. 2020;8: 378. doi:10.3390/microorganisms8030378

9. Ferrières L, Hémery G, Nham T, Guérout A-M, Mazel D, Beloin C, et al. Silent mischief: bacteriophage Mu insertions contaminate products of Escherichia coli random mutagenesis performed using suicidal transposon delivery plasmids mobilized by broad-host-range RP4 conjugative machinery. J Bacteriol. 2010;192: 6418–6427. doi:10.1128/JB.00621-10

10. Kaniga K, Delor I, Cornelis GR. A wide-host-range suicide vector for improving reverse genetics in gram-negative bacteria: inactivation of the blaA gene of Yersinia enterocolitica. Gene. 1991;109: 137–141. doi:10.1016/0378-1119(91)90599-7

11. Lutz R, Bujard H. Independent and Tight Regulation of Transcriptional Units in Escherichia Coli Via the LacR/O, the TetR/O and AraC/I1-I2 Regulatory Elements. Nucleic Acids Res. 1997;25: 1203–1210. doi:10.1093/nar/25.6.1203
