# Supplementary material for: Interplay between the cell envelope and mobile genetic elements shapes gene flow in populations of the nosocomial pathogen Klebsiella pneumoniae
Source: PLoS Biol. 2021 Jul 6;19(7):e3001276. doi: 10.1371/journal.pbio.3001276 (PMC8259999; doi:10.1371/journal.pbio.3001276)
Supplement: Attachment — Submitted filename: Response.pdf [file pbio.3001276.s010.pdf]

Matthieu Haudiquet
Microbial Evolutionary Genomics Lab
28 rue du Docteur Roux, 75015 Paris, France
Tel: +33 1 40 61 36 37
Email: matthieu.haudiquet@pasteur.fr

Paris, 4/10/21

Subject: Submission of a revised manuscript

Dear Dr. Jauregui,

Thank you for editing our manuscript and inviting us to revise it in consideration of the reviewer's comments. We thank all three reviewers for the relevancy of their reviews and have implemented their suggestions in the manuscript. We answered all their comments below, in each case explaining how we have revised the manuscript in light of the comment. Here, we briefly mention the key issues.

As requested by Reviewer #1, we added appropriate references evidencing similar capsule inactivation under phage pressure and in the condition of our evolution experiments. We agree with Reviewer #1 that depolymerase similarity between prophages infecting the similar capsule serotype is likely to explain some of the genetic similarity between these prophages. However, we lack a robust method to annotate them and now mention other works that have recently highlighted this annotation problem.

As requested by Reviewer #2, we added a phylogenetic tree to show the scattered distribution of the non-capsulated isolates. We implemented a correction by gene length in our analysis of the capsular pseudogenes, which was added in the supplemental material, and gives similar qualitative conclusions. Finally, we have explained in more detailed how we checked that pMEG-Mob was unlikely to be mobilized in *trans* by the Kpn strains.

As requested by Reviewer #3, we clarified the cut-offs used to type the capsule locus. We now include one table with the mutations found in the lab evolved clones and another with the information on the pseudogenes identified in natural isolates.

These changes clarified some important points of our study, but led to no significant change of our conclusions. We hope you will find that we have effectively addressed the comments of the reviewers and your own, and that our improved manuscript is now suitable for publication in PLOS Biology.

Best regards,

Matthieu Haudiquet

Answers to reviewers

Reviewer #1:

1.In this manuscript, Haudiquet and colleagues bioinformatically analyse genome sequencing data to investigate the relationship between horizontal gene transfer (HGT) and capsule serotypes in the important opportunistic pathogen Klebsiella pneumoniae. They find an increased incidence of HGT within, rather than between, predicted serotypes. This they ascribe to transduction as a consequence of phage activity, which is often serotype-specific. They then investigate serotype swaps, which occur by recombination, probably with a brief intermediate uncapsulated state, which they experimentally show emerges readily in the laboratory by mutation. Capsule inactivation is associated with an excess of HGT, both by bioinformatic analyses, and increased conjugation efficiency in the lab, which can result in re-acquisition of a capsule. Together this analysis shows the state of flux in capsule presence, and how this process might structure gene exchange more broadly.

Overall I really enjoyed reading this paper — not only did it draw a dynamic and compelling (co)-evolutionary picture from comprehensive and sophisticated bioinformatic analyses, but it provided valuable experimental validation for several of the key mechanisms posited. As Klebsiella is a major clinical concern, I anticipate that the findings extend beyond evolutionary microbiology and will have broader relevance to genomic epidemiology. I have only a few comments and suggestions for the authors to consider in any revised submission.

Answer: We thank the reviewer for the positive assessment of the manuscript and helpful comments which have improved the quality of the manuscript.

2.- The proposed evolutionary process depends on phage predation to select for loss of the capsule, but the lab-evolved uncapsulated strains were evolved in LB media (and so were presumably under selection for capsule loss due to the metabolic cost of production). What evidence is there that capsule inactivation evolves in a similar manner under phage pressure?

Answer: In a previous work, we performed a similar evolution experiment to the one presented here, and we precisely showed that capsule inactivation is accelerated under phage pressure (de Sousa et al. 2020). Further, other recent publications have shown that phage pressure leads to the loss of the capsulated phenotype by mutations in the same genes (especially *wcaJ,* Glycosyl transferases and *wzc*) in phage-resistant *K. pneumoniae* (*Kpn*) clones. Interestingly, while this article was in review, a study on essential genes in *Acinetobacter* found that inactivation of the latter steps in the capsule biosynthesis pathway was extremely deleterious, whereas the loss of the genes for the initial steps was tolerated (Bai et al, 2021). Since growth in LB and under phage pressure seem to select for capsule loss, we expect similar patterns of genetic inactivation.

We have added details and references (Hesse et al, 2020, Tan et al, 2020, de Sousa et al 2020) in the manuscript supporting that capsule inactivation evolves in a similar manner under phage pressure and the condition of our evolution experiment. We also discuss this point more clearly.

3.- Line 137 looks for similarities between prophages of strains with similar capsule compositions and finds a weak proportionality. Would this relationship be strengthened by looking specifically at similarities between the depolymerase genes of those phages, rather

than the phages as a whole? What evidence is there for depolymerase gene exchange between different phages, in a reciprocal coevolutionary manner to capsule switches?

Answer: We agree that similarity between prophages of strains with similar capsule composition may be driven to some degree by the similarity of the depolymerase genes of those phages. We previously tried to annotate such genes (de Sousa et al, 2020), without success, as homologs of known depolymerases do not match any gene present in our prophage dataset. Similar observations that many phages in *Kpn* lack known depolymerases, even if the capsule is present in most strains, have recently been done by other groups (M Townsend et al, 2021). In the light of this and lack of a validated methodology to annotate such proteins, we could not test this hypothesis.

We agree with the expectation that there may be depolymerase swaps between phages, as a reciprocal phenomenon to capsule serotype swaps. We have now included this hypothesis in the discussion.

Minor comments and suggestions:

4.- Figure 1E shows a summary of the means calculated across millions of points. Error bars or the full data should be presented (in supplementary information if not in the main figure).
Answer: We have now added error bars in the main figure.

5.- Line 85 states that the capsule *needs* MGEs to vary by HGT. The authors should explain here why transformation is not considered a viable route.
Answer: As far as we know, natural transformation has never been observed in *K. pneumoniae*. We have added this information in the manuscript to explain why we focus on conjugative elements and temperate phages.

6.- The sentence line 87-88 is not clear ('its').
Answer: We replaced “its” by “the capsule’s”

7.- Somewhere in the paragraph starting line 317, the authors should make clear that capsule inactivation results in higher rates of gene *acquisition* by conjugation (rather than gene donation by conjugation). Does capsule presence affect outward conjugation?
Answer: We have clarified that the conjugation rate is increased on the recipient end. The impact of the capsule on outward conjugation is a very interesting point, but cannot be tested with the same experimental protocol, where the donor is always the same. Our preliminary data on a very different setup suggests that the status of the capsule of the donor also has an effect (which is independent of the effect of the recipient). However, it’s too soon to add that data to this work (and that would significantly lengthen the manuscript). Also, our focus here, in both the experimental and computational analyses, is on recipients, not donors. The effect of the capsule on donors will be the subject of a future publication.

8.- Figure 6 — state that conjugation efficiency is T/N in the legend.
Answer: We have added this information on the y-axis and in the legend.

9.- Figure 6. What is the known fitness benefit/cost of the non-capsulated mutants? This is unlikely to have a major effect on the outcome given the short period provided for conjugation, so perhaps the authors could give readers peace-of-mind by mentioning the short conjugation period either in the text or in the legend.
Answer: The growth rate difference between non-capsulated and WT capsulated clones has been investigated in Buffet *et al.*, 2021, where the frequency of non-capsulated clones was at most 20% higher than that of non-capsulated clones (in shaking liquid cultures in LB) after

24h. As strains were placed on the conjugation membrane for only one hour, growth rate differences cannot explain the different amount (up to 20-fold) of transconjugants we observed.

We added details in the results section stating that the conjugation period was too short for growth rate differences to justify the observed differences.

10.- Figure S3. The panels are the wrong way round.
Answer: This has been corrected.

11.- Please check data availability box (accession numbers still read 'XXX').
Answer: We have checked the availability of the genomes and submitted three that were not yet available (208/210/212).

Reviewer #2:

1. In this work, Haudiquet et al. nicely explore the complex relationship between the capsule and HGT in Klebsiella pneumoniae (kpn). Through a combination of experiments and bioinformatic analyses, they convincingly show that phages typically infect kpn strains with same capsule type, whereas conjugative elements spread regardless of capsule type. Phages also drive capsule inactivation, which in turn increases the overall acquisition of conjugative elements, as the capsule hampers conjugative DNA transfer. Overall, the manuscript is well-written, the analyses are well-thought and statistically supported, the results are important and novel and will be of great interest to the broad audience of PLoS Biology. I only have some minor comments regarding data presentation and some experimental issues.

Answer: We thank the reviewer for the positive comments and careful review which have helped improving the manuscript.

2.- I found the introduction part describing capsule loci and the capsular locus types a little bit confusing (L57-62). The authors might want to include here the notion that Kpn capsule is encoded in the galF-ugt locus, and improve CLT definition to state what are the differences among distinct CLTs. In addition, thorough the manuscript the authors refer to CLT and serotype interchangeably. If both terms are really the same, I would suggest to stick to one of them for the sake of simplicity.
Answer: We have clarified the description of the capsular locus, included the notion that it is located between *galF* and *ugd*, and improved the definition of the CLT. We specifically mention that both terms are not synonyms, and corrected the cases in the manuscript that could confuse the reader.

3.- Figure 1A legend. The authors should state that the tree is a core genome tree of Kpn.
Answer: We have added this missing information

4.- L109. I think the authors mistakenly refer to figure 1C instead of 1D?
Answer: This has been corrected.

5.- L120. The authors should add one or two sentences to explain a bit more wGRR, as it is a central parameter for their analyses. How should a non-expert interpret this parameter?
Answer: The wGRR is a comparison of the gene repertoire between two sequences, weighted by their genetic similarity. It amounts to zero if there are no homologous proteins between the genomes, and one if all genes of the smaller genome have an identical

homolog in the other genome. Hence, the wGRR accounts for both frequency of homology and degree of similarity among homologs.
We have clarified the material and methods section about the wGRR and added a sentence explaining it in the results section.

6.- Figure 1E. Why the data plotted only covers a small range of prophage wGRR (0.08-0.105), whereas in figure 1C it ranges from 0.2-1? Also, there seems to be some small dots on the plot that are not represented on the scale.
Answer: We changed the graph presented in figure 1D (previously 1C) to represent the complete range of the data. The frequencies are now computed with the full range of the data. They were previously computed from the presented subset wGRR>0.2 (hence the change in scale) to highlight the key region (high wGRR values). We shifted the bins from [0,0.1[to]0,0.1] to better represent the sharp increase of similar prophages around the]0,0.1] bin. This part of the graph is now zoomed-in as an inset.
The scale of the graph on figure 1E is simply the complete range of the data presented. We have also added error bars around the points (+/-SEM).

7.- Figure 3A. Perhaps the figure y-axis could be changed to show the frequency instead of the absolute count, to better relate with the text (which states percentage of loci).
Answer: We have changed the y-axis to frequencies to better relate with the text.

8.- L186. Although it is not essential for the main message of the manuscript, the authors could look at whether the galF-ugd locus is more prone to engage in recombination (i.e. hyper-recombinogenic) in comparison to other parts of the genome or the overall recombination rate of the species.
Answer: The *galF-ugd* locus is indeed more prone to engage in recombination than the rest of the genome. We did not include this analysis as this was previously extensively shown for *K. pneumoniae* (and other species like *Streptococcus pneumoniae*). We have added this information with appropriate references in the manuscript.

9.- L209. I would suggest the authors to show a phylogenetic tree indicating the branches with inactivated capsules as a supplementary figure, to better represent the idea that there's no phylogenetic signal.
Answer: We have added an annotated tree as a supplementary figure showing the phylogenetic distribution of the inactivated capsules. (Figure S3)

10.- L229. Other interpretations are also possible. For instance, it may well happen that mutations in the first steps are more likely to occur (i.e. highly variable locus, homopolymeric tracts, insertion sequences). Although it is highly unlikely to affect the results, the size of the mutational target should also be taken into account. In addition, Do the authors see any repeatability in the type of mutations isolated in the evolution experiment? I do believe that the authors should provide a supp. table indicating which specific mutations were found on the evolution experiment.
Answer: In response to this comment, we have added details in the main text and a new supplementary figure (Figure S4) which contains:

- A correction of the inactivation frequency by the length of the genes, which does not change the relationship shown on figure 3B.
- An analysis of the content in homopolymer tracts and dinucleotide simple sequence repeats (SSR) in the capsule core genes (including the ones on Figure 3B), which show that their abundance varies according to the gene. Given the genes in Figure 3B, the SSR in *wcaJ*/*wbaP* are less abundant than in *wzx*/*wzy*/*wzc* and should thus not explain our results.

- An analysis of the allelic diversity of the core genes (including the ones on Figure 3B) as measured by their percentage of identity for all pairwise alignments, which shows that *wcaJ*/*wbaP* are not the most diverse genes of the capsule locus.
- A table of all mutations found by *snippy*, *breseq*, and our approach in the evolved clones we sequenced (Dataset SD3).

We also discuss the difficulty of detecting IS in draft assemblies in the "Handling of draft assemblies" section of the methods, since they will often result in contig breaks. We do not observe repeatability in the sense that clones do not share the exact same mutation at the base pair level. These analyses do not suggest that our hypothesis, selection for inactivation of early genes in the biosynthetic pathway, is affected by the existence of mutation hotpots in the locus.

11.- L707-710. Most of the strains carry other plasmids apart from the pMEG-Mob (e.g. strain 342 has 6 plasmids). Have the authors checked that none of these plasmids are able to mobilize in trans the pMEG-Mob? Were appropriate controls included in the conjugation experiments? If so, it should be indicated.

Answer: We verified that the strains used in the conjugation assays did not code for a conjugative system of type MPF type I, the one of or RK2, which is the one able to mobilize pMEG-Mob. Moreover, we compared the conjugation efficiency always from the same strain, which would have controlled for the mobilization of pMEG-Mob by any resident conjugative system.

We have added this information in the results and methods.

12.- I found really interesting that the main determinant of capsule swap is chemical composition and not genome similarity, which is what one would expect for recombining DNA fragments. The authors could discuss this further.

Answer: We agree and have now added this topic in the discussion.

Reviewer #3:

1. This is an interesting paper which provides some evidence that strains of Klebsiella pneumoniae (Kpn) pass thhrough a stage of non-capsulation that might make them more susceptibe to HGT, especially by conjugative plasmids. Conjugation is likely to mediate examples of capsule switching they observe in their genomic data, which will alter the propensity of strains to be infected by capsule-recognising phages. Clearly the authors present data based mainly on bioinformatic analysis rather than experimentation (excpet for some conjugation experiments) so they need to be cautious drawing direct caasal links between sequence data and what happens during the natural lifecycle of Kpn.

Answer: We thank the reviewer for the suggestions and corrections provided. Since we mainly present the results of computational biology analysis, we have toned down some sentences drawing direct causal links between our findings and the natural lifecycle of Kpn.

Specific comments

2. Data Fig 1 C refers to prophages while the text doesn't (line 107, We found significantly more genes acquired (co-gained) in parallel by different isolates having the same CLT than expected by simulations assuming random distribution in the phylogeny (1.95x, Z-test p<0.0001, Figure 1C), and it is not clear where these data are in the Figure.

Answer: This has been appropriately modified.

3. The authors should be claer about what their cut-offs are for deciding which capsules are similar and which are different. This is not provided in the results or materials and methods.
Answer: We set a quality threshold for the prediction of the CLT, which we explain in more details in our answer to comment 10. After that, we consider that two isolates have the same CLT if the predicted CLT is the same. We have replaced “similar serotypes” by “chemically similar serotypes”, that is, share common residues, when necessary.

4. 136 The genomes with these CLTs, 59% of the total, show a weak but significant proportionality between prophage similarity and the number of similar residues in their host capsules(Figure 1E)..
Please provide details
Answer: We are not certain of which details would be required to satisfy this request. Following reviewer #2, we have added error bars to the graph.

5. Please provide information about which strains did you select for evolution experiments and why? What are their capsule groups/phylogeny etc and did you recapitulate any mutations found in the epidemiologic collection…
Answer: The evolution experiment was designed to be representative of the genetic diversity of the *Klebsiella pneumoniae* species complex, in terms of source of isolation, capsule serotype and ST. The strains analyzed here represent eight different Sequence Types (as determined by Kleborate), 6 different capsule locus types, and 6 different isolation sources and geographical origins.
The information on the strains is provided in Table S2 and the diversity of the sample is now quickly described in the main text.

Since our pseudogene detection approach relies heavily on tblastn, predicting the precise mutation leading to the inactivation is not straightforward. Indeed, tblastn can sometimes extend the alignment by a few nucleotides. Hence, we cannot systematically assess the exact position of frameshifts, stop mutations and small deletions. Moreover, Kaptive was used to determine the absence of expected genes in the case of complete gene deletion, and this approach does not lead directly to genomic positions of mutations. Hence, we recapitulated the inactivated ORFs on figure 3C, and their frequency of inactivation in the genomic dataset can be seen on figure 3B.

6. L340 Explain what the Conjugation efficiency is here please: Show the strain serotypes here please.
Answer: Explanations are now given in Methods (efficiency was replaced by frequency, which is more accurate). We have added this information to the graph.

7. These are passaged strains so might harbor other mutations. Is this the case? You have Illumina sequencing data. The changes vs the parental strains should be given as supllementary data.
Answer: Most strains only have one mutation. This is not unexpected as the strains evolved only 20 generations and it’s the strength of selection process that leads to the high frequency of capsule loss in the population. We now provide a table with all mutations identified (supplementary dataset 3).

8. The branch length is much longer in strains with capsule swap (Fig S4) so I do not think that it is valid to include this group of strains in Fig 4 A and B. This might explain why there is evidence of more gene gain when there has been a swap rather than when there is capsule inactivation. Furthermore, are the events leading to swaps themselves discounted from

these analyses? Although authors acknowledge this on line 268, the branches where capsules were swapped are 2.7 times longer than the others, precluding strong conclusions (Figure S4). So these data should be exclude from the main results section.

Answer: There are two different points that are made with this figure.

Firstly, branches where there are changes in the capsule (inactivation or swap) have more changes in terms of gene gain. Concerning capsule inactivation, there is no significant difference in branch lengths to the others nor are recombination tracts particularly expected at the capsular locus. Therefore, the comparisons are fair and meaningful even without correction. As for swaps, it is indeed the case, as we noticed that branches are 2.7 times longer. But the effect observed clearly exceed this factor (4.5 vs 6.5). Hence, a linear correction by branch length would still reveal a difference to branches where the capsule remained constant. We agree that the branch length being different, caution should be taken when interpreting this value. Indeed, the isolates which underwent a recent swap have lengthy recombination tracts (Figure 3C), which may increase branch lengths in phylogenies. We could not infer the recombination tract of every genome associated with a swap, so we did not discard these events from the analysis.

Secondly, there are more prophages acquired than conjugative elements on branches with a swap, whereas it is the opposite trend for branches with a capsule inactivation. These comparisons are made for the same sets of terminal branches and the fact that swapped branches are longer does not affect the analysis.

We have restructured this part of the text to precise that the comparison should not be between inactivated and swapped directly, but more on the relative acquisitions of MGEs.

9. Also it is important to include in the discussion of the paper that it is possible that changes leading to capsule inactivation could arise during passage in vitro before sequencing and necessarily occur not in natural populations.

Answer: We agree this needs more discussion and have now added it to the text. We cannot exclude that some mutations got fixed in the lab. However, passaging for sequencing provides little opportunity for such mutations to fixate. Only those mutations under very strong positive selection would be fixed. We observe that 1) many isolates have several pseudogenes within their capsule locus (figure 3A), 2) many gene inactivations are associated with non-essential genes, which are not expected to be under strong selection. and 3) had we counted these inactivations, the frequency of non-capsulated clones would almost triple. These results suggest that many of the mutations accumulated over time and not during passage. One would also like to emphasize that while it's common to sustain that *Kpn* are almost always capsulated, we know of no work showing that it is the case for more than 97% of the strains. On the other hand, there are multiple reports of non-capsulated strains with increased biofilm formation and epithelial cell invasion recovered from patients with persistent urinary tract infections (Ernst et al, 2020)

10. 98 How was this done? Around 92% of the isolates could be classed with good confidence-level.

Answer: We used Kaptive to infer the capsule locus type of each genome assembly. Kaptive relies on a series of blast against the Klebsiella K locus reference database, with the whole loci (blastn), and each gene (tblastn). A score is computed from the results of the alignments, the gene content and the fragmentation of the capsule locus. We used the threshold recommended by the authors of the software.

Genomes with CLTs with a confidence level below "good" were excluded from all the analysis using the CLT as a variable. This is same threshold used by the authors of the software.

11. 143 Klebsiella quasipneumoniae subsp. similipneumoniae (Kqs) explain please here?
Answer: We added the information (previously only in the methods) that these strains are wrongly annotated as *Kpn* (certainly because they are a recent subspecies, that is very close genetically to Kpn) in the RefSeq dataset, and formed a natural outgroup to our phylogenetic tree.

12. 161 expected from previous works
Answer: We have modified this

13. 165 implicates. Better suggests
Answer: We have changed to imply. We believe that since the genes are radically different upon swap this is clear evidence for HGT. Suggest does not convey enough the association we want to make.

14. 216 which strains did you select for evolution experiments and why? What are their capsule groups/phylogeny etc and did you recapitulate any mutations found in the epidemiologic collection…
Answer: This is an important point that is answered above (Comment #5).

15. 262 acquired in the branches present (not acquired) …
Answer: In this context, we specifically refer to the mobile genetic elements that have been acquired in the time period represented by the branches.

16. 292: conjugative systems
Answer: Systems was replaced by elements.

17. 320 in the absence of published data on the frequency of conjugation in function of the presence of a capsule. Please check this sentence.
Answer: We have corrected this sentence.

18. I am struggling with Figure 5 see what has been calculated. Please can you add further text to explain.
Answer: We have added additional text in the legend of Figure 5 and in the methods section to clarify what has been calculated and the interpretation of the result.

19. 364 more than wild type rather than other…
Answer: We have corrected this sentence.

20. 379 There are multi-drug resistant hyper-virulent clones. Do you see evidence of capsule switches in these strains, which would be consistent with them experiencing a period of loss of capsule (when they could acquire the resistance plasmid) followed by restoration of capsule expression by conjugation.

Answer: This is a very interesting point that we could not deepen in our manuscript due to lack of enough genomic data. Multi-drug resistant hyper-virulent clones are still rare, and so are their genome sequences in public databases.
However, during the time of revision of this manuscript, a new genome of a Spanish strain harboring a convergent resistance / virulence conjugative plasmid was published (Hernández et al, 2021). We have analyzed the capsule locus of this strain, and found that it is heavily degraded. Here is a comparison with the reference serotype KL1, closest match from the new strain:

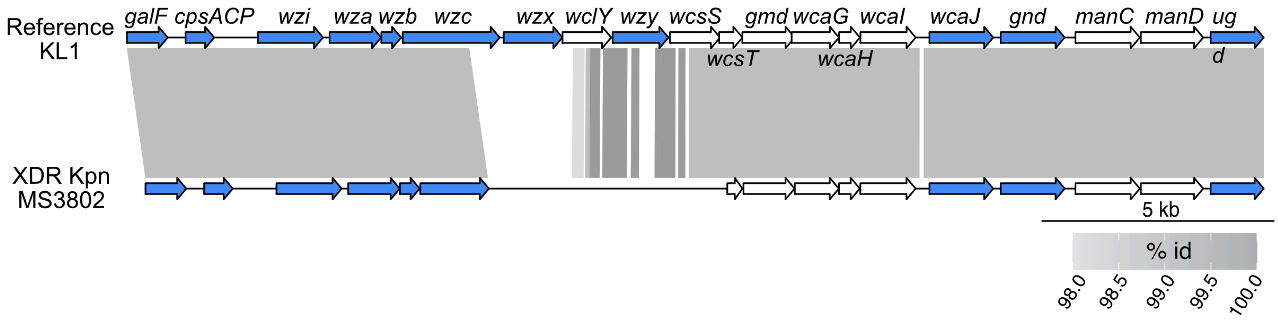

This could suggest that capsule inactivation facilitated the acquisition of a conjugative, resistance plasmid, which later fused with the resident virulence plasmid.
We have added this information in the discussion.

21 Figure 7 could be simplified.
I do not think that both panels are necessary:
Fewer bacteria in panel A would be less confusing… just use bacteria which are subject to the relevant steps. And in B, please colour the cap locus with the appropriate colour to highlight what is being transferred during conjugation…
Answer: We have removed some cells from panel A, however left a few of them to communicate the idea that we are focusing on population of cells. We have colored the capsule locus with the color of the serotype in the panel B

COMMENTS FROM THE ACADEMIC EDITOR:

In addition to the points raised by the reviewers, the authors may want to clarify that variation in cell density of WT and non-capsulated versions of the strains used in the conjugation assays (Fig. 6) cannot explain the higher conjugation efficiency of non-capsulated strains. Cell density possibly affects mating opportunities in their filter matings beyond what can be controlled for by the ratio of transconjugants to total cell number. A statement that cell densities of WT and non-capsulated strains were not systematically different would suffice.

Answer: Cell densities were normalized to the same OD_{600} (OD=0.9) before mating, and the donor culture was the same for all recipient strains per biological replicate. Yet, following the editor's suggestion, we looked in more detail at the total number of cells that were plated. We found that there were fewer CFU on the filters with non-capsulated cells than with the WT (3.2x less, t-test $p<0.001$). This can be due to the fact that OD_{600} and CFU do not follow the same correlation for capsulated and non-capsulated strains. Hence, the density of cells was slightly lower in the experiments of conjugation for non-capsulated mutants than for the WT. Lower cell density usually results in lower conjugation frequencies. Yet, we found the opposite (by an order of magnitude higher). Hence, we conclude that the conjugation rate of the non-capsulated mutants is likely a slight underestimate of the true value, which does not change the conclusion of the experiment.
We have added this information to the result part of the manuscript.
