# Supplementary material for: Interplay between the cell envelope and mobile genetic elements shapes gene flow in populations of the nosocomial pathogen Klebsiella pneumoniae
Source: PLoS Biol. 2021 Jul 6;19(7):e3001276. doi: 10.1371/journal.pbio.3001276 (PMC8259999; doi:10.1371/journal.pbio.3001276)
Supplement: Attachment — Submitted filename: HGT_Serotype_Answers_SecondRound_final.pdf [file pbio.3001276.s011.pdf]

Answers

1. We recommend a change in the title to include the pathogen: "Interplay between the cell envelope and mobile genetic elements shapes gene flow in populations of the nosocomial pathogen Klebsiella pneumoniae."

We have changed the title to include the pathogen.

2. Note that we do not require all raw data. Rather, we ask that all individual quantitative observations that underlie the data summarized in the figures and results of your paper be made available in one of the following forms:
1) Supplementary files (e.g., excel). Please ensure that all data files are uploaded as 'Supporting Information' and are invariably referred to (in the manuscript, figure legends, and the Description field when uploading your files) using the following format verbatim: S1 Data, S2 Data, etc. Multiple panels of a single or even several figures can be included as multiple sheets in one excel file that is saved using exactly the following convention: S1_Data.xlsx (using an underscore).
2) Deposition in a publicly available repository. Please also provide the accession code or a reviewer link so that we may view your data before publication.

We have deposited all the datasets in Figshare with the appropriate convention:
http://doi.org/10.6084/m9.figshare.14547324

3. Regardless of the method selected, please ensure that you provide the individual numerical values that underlie the summary data displayed in the following figure panels as they are essential for readers to assess your analysis and to reproduce it: Figures 1B, 1C, 1D, 1E, 2A, 2C, 3A, 3B, 3C, 4A, 4B, 5, 6, Supplementary figures 2A, 2B, 4A, 4B, 4C, 5A, 5B, 6, 7.
NOTE: the numerical data provided should include all replicates AND the way in which the plotted mean and errors were derived (it should not present only the mean/average values). Please also ensure that figure legends in your manuscript include information on where the underlying data can be found, and ensure your supplemental data file/s has a legend.

We have added corresponding datasets (numerical values for all the replicates and simulations) for all the figures cited, and their name in the figure legends.

4. Please ensure that your Data Statement in the submission system accurately describes where your data can be found.

We have updated the Data statement so that all the publicly available sequences and our deposited datasets can be found.
